# Epilepsy and Neurocysticercosis in Latin America: A Systematic Review and Meta-analysis

**DOI:** 10.1371/journal.pntd.0002480

**Published:** 2013-10-31

**Authors:** Elisa Bruno, Alessandro Bartoloni, Lorenzo Zammarchi, Marianne Strohmeyer, Filippo Bartalesi, Javier A. Bustos, Saul Santivañez, Héctor H. García, Alessandra Nicoletti, the COHEMI Project Study Group

**Affiliations:** 1 Department G.F. Ingrassia, Section of Neurosciences, University of Catania, Catania, Italy; 2 Department of Critical Care Medicine and Surgery, Infectious Diseases Unit, University of Florence, Florence, Italy; 3 Cysticercosis Unit, Instituto Nacional de Ciencias Neurologicas, and Center for Global Health - Tumbes and Department of Microbiology, Universidad Peruana Cayetano Heredia, Lima, Peru; George Washington University, United States of America

## Abstract

**Background:**

The difference in epilepsy burden existing among populations in tropical regions has been attributed to many factors, including the distribution of infectious diseases with neurologic sequels. To define the burden of epilepsy in Latin American Countries (LAC) and to investigate the strength of association with neurocysticercosis (NCC), considered one of the leading causes of epilepsy, we performed a systematic review and meta-analysis of the literature.

**Methodology:**

Studies published until 2012 were selected applying predefined inclusion criteria. Lifetime epilepsy (LTE) prevalence, active epilepsy (AE) prevalence, incidence, mortality, treatment gap (TG) and NCC proportion among people with epilepsy (PWE) were extracted. Median values were obtained for each estimate using random effects meta-analysis. The impact of NCC prevalence on epilepsy estimates was determined using meta-regression models. To assess the association between NCC and epilepsy, a further meta-analysis was performed on case-control studies.

**Principal findings:**

The median LTE prevalence was 15.8/1,000 (95% CI 13.5–18.3), the median AE prevalence was 10.7/1,000 (95% CI 8.4–13.2), the median incidence was 138.2/100,000 (95% CI 83.6–206.4), the overall standardized mortality ratio was 1.4 (95% CI 0.01–6.1) and the overall estimated TG was 60.6% (95% CI 45.3–74.9). The median NCC proportion among PWE was 32.3% (95% CI 26.0–39.0). Higher TG and NCC estimates were associated with higher epilepsy prevalence. The association between NCC and epilepsy was significant (p<0.001) with a common odds ratio of 2.8 (95% CI 1.9–4.0).

**Significance:**

A high burden of epilepsy and of NCC in LAC and a consistent association between these two diseases were pointed out. Furthermore, NCC prevalence and TG were identified as important factors influencing epilepsy prevalence to be considered in prevention and intervention strategies.

## Author Summary

Epilepsy affects approximately 70 million people worldwide and at least five million people in Latin America. Many researchers have pointed out a different distribution of epilepsy in Latin American countries, with some regions presenting higher frequencies and others presenting lower frequencies. This difference in epilepsy distribution has been attributed to many factors, mainly related to the allocation of health resources and to the presence of environmental and infectious risk factors. Among the latter stands neurocysticercosis, a parasitic disease that has been recognized as the leading cause of acquired epilepsy in the developing world, with a particularly elevated distribution in rural settings. In this study, we performed a statistical analysis to investigate whether neurocysticercosis distribution affects epilepsy distribution among Latin American countries and the relationship between these two conditions. The combined results of the studies included indicated that neurocysticercosis influences epilepsy frequency in Latin America, as countries with higher epilepsy distribution presented also higher neurocysticercosis frequency. Moreover, another analysis pointed out an association between the two diseases. These results appear very important considering that parasitic infections are modifiable factors and that their reduction may contribute to decrease epilepsy burden worldwide.

## Introduction

Epilepsy is one of the most prevalent non-communicable neurologic diseases [1], with an estimated aggregate burden of around 0.5% of the total disease burden [2]. It affects approximately 70 million people worldwide [3] and at least five million people in Latin American Countries (LAC) [4].

The epidemiological studies describing the burden of epilepsy across the world have frequently reported the presence of important differences in the estimate of prevalence and incidence [5]. The median lifetime epilepsy (LTE) prevalence, ranges from 5.8/1,000 (range 2.7–12.4) in developed countries to 15.4/1,000 (range 4.8–49.6) in rural areas of developing countries [3], and similar variations are also reported for active epilepsy (AE) prevalence and for incidence [6]. Considering LAC, the median LTE prevalence ranges from 6/1,000 to 43.2/1,000, while the median AE prevalence from 5.1/1,000 to 57/1,000 [7], showing a very large range of variability.

The wide prevalence difference existing among populations may be mainly attributed to country resources and development-related factors [8] to spatial clustering of etiologic and risk factors [9], and to methodological limitations of studies [10]. In a recent review on the global burden of epilepsy, a meta-regression analysis showed that *location*, *study size* and *age of study participants* explained 53% of the variance in LTE prevalence [3].

However among the factors probably responsible of the unexplained amount of variance, the distribution of epilepsy-related biological and environmental factors, such as infections of the central nervous system (CNS), may be important, especially in resource limited countries, but had never been taken into consideration in previous meta-analysis.

Among CNS infections, neurocysticercosis (NCC) is considered the leading cause of acquired epilepsy in the developing world [11], [12]. Although it has been declared eradicable by the International Task Force for Disease Eradication of World Health Organization (WHO) in 1993, NCC is still recognized as a “major neglected disease” due to the lack of information about its burden and transmission, the lack of diagnostic tools available in resource-poor areas, and the lack of intervention strategies for its control [13]. Recent data indicate that NCC represents a significant health problem in endemic areas, causing epilepsy in 0.6–1.8% of the population [13]. This indicates that between 450,000 and 1.35 million persons suffer from epilepsy due to NCC in LAC only [14].

Understanding the reasons that influence epilepsy distribution is crucial to improve and tailor intervention programs and prevention strategies. Thus, to better define the burden of epilepsy of NCC and their association in LAC, we conducted a systematic review and meta-analysis of epilepsy prevalence, incidence, mortality, treatment gap (TG) and of NCC prevalence among people with epilepsy (PWE) in LAC.

## Methods

### Search strategy

Two systematic searches, without language restriction, were conducted to identify all relevant articles concerning “burden of epilepsy” and “prevalence and association between cysticercosis (CC)/NCC and epilepsy”. The following electronic databases were independently examined by two authors (EB and JB) to identify articles published until the 1^st^ July, 2012: MEDLINE, IMBIOMED, LILACS, EMBASE, SciELO, PAHO Library Online Catalog, PAHO Evidence Portal, WHOLIS, Cochrane Library. Additional searches were performed on bibliographies of pertinent original articles, reviews, abstracts and book chapters. Combined text words and Medical Subject Headings (MeSH) terminology were used. Searches were organized using the following search terms to develop a search strategy: “epilep*” and “mortality”, “ preval*”, “incidenc*”, “epidemiol*”, “ surve*”, “rate*”, “frequenc*”, “treatment gap”, “cysticerc*”, “neurocysticerc*”, “taenia*”, “Argentina”, “Bolivia”, “Brazil”, “Chile”, “Colombia”, “Costa Rica”, “Cuba”, “Dominican Republic”, “Ecuador”, “El Salvador”, “Guatemala”, “Guyana”, “Honduras”, “Mexico”, “Nicaragua”, “Panama”, “Paraguay”, “Peru”, “Puerto Rico”, “Suriname”, “Uruguay”, “Venezuela”, “Latin America”. The literature search was adapted for the different databases.

### Selection criteria

#### Burden of epilepsy

We included retrospective cross-sectional or prospective studies measuring prevalence, incidence, mortality or TG of epilepsy in adults or children from LAC.

A paper was included if it reported a definition of epilepsy as two or more unprovoked seizures occurring at least 24 hours apart [15], if it included an accepted definition of LTE and AE [15], and provided the denominator of the population. We also included studies published before 1993 if a definition of epilepsy, comparable with those above-mentioned, was provided. Regarding TG, the studies meeting the definition proposed by the International League Against Epilepsy [16], were included. Data should have been collected through standardized questionnaires in door-to-door survey. A study was excluded if it explored only acute symptomatic seizures or specific seizure patterns or epileptic syndromes. Data from reviews, editorials, abstracts, letters to the editor and studies of subpopulation were excluded.

#### NCC prevalence among PWE

Retrospective cross-sectional or prospective studies measuring the prevalence of NCC among PWE in LAC were included if they reported a definition of epilepsy, the description of ascertainment methods of epilepsy, NCC diagnostic methods (reported below), and study population: rural/urban, community/hospital (inpatient/outpatient).

The diagnosis of NCC is currently based on a set of criteria including clinical evaluation, radiological, immunological and epidemiological data, that results in two categories of diagnostic certainty: definite and probable NCC [17]. We included studies in which the diagnosis of NCC met the definition of probable or definite according to the above mentioned criteria [17]. Therefore, we included studies combining neuroimaging, both brain computed tomography (CT) scan or magnetic resonance imaging (MRI), to serum enzyme-linked immunoelectrotransfer blot (EITB) assay, whose specificity approaches 100% and sensitivity 94–98% for patients with two or more cystic or enhancing lesions [18]. Studies detecting anticysticercal antibodies by cerebrospinal fluid (CSF) enzyme-linked immunosorbent assay (CSF AbELISA) were also included [19]. Studies using only serum antibody enzyme-linked immunosorbent assay (AbELISA) or serum antigen enzyme-linked immunosorbent assay (AgELISA) were not included [20].

#### Association between CC/NCC and epilepsy

Studies meeting the following criteria were included in the meta-analysis: presence of a control group (people without epilepsy, PWOE); information about methods and criteria used for case-finding and control selection; possibility to determine the sample size of each of the following four groups: PWE affected by CC (PWE CC+), PWE not affected by CC (PWE CC−), PWOE affected by CC (PWOE CC+), PWOE not affected by CC (PWOE CC−), presence of a valid diagnostic method to assess CC/NCC (either brain CT, brain MRI, serum EITB, or CSF AbELISA). Serum AbELISA and serum AgELISA were not considered valid diagnostic methods for the frequently reported false positive results with the first and false negative results with the second technique [21].

### Data extraction

Two reviewers (EB and JB) independently assessed the titles and abstracts of all the studies identified. The full copies of papers requiring further consideration were obtained. Relevant studies were selected according to the criteria outlined above and data were independently extracted on a predefined collecting form.

### Statistical analysis

#### Burden of epilepsy and of NCC among PWE

Crude prevalence was expressed as the number of cases per 1,000 people. We recalculated the 95% confidence intervals (95% CI) around the estimates provided. Crude incidence estimates were expressed per 100,000 persons per years +/−95% CI. Mortality estimates were reported using standardized mortality ratios (SMR) +/−95% CI. Magnitude of the TG was expressed as a percentage +/−95% CI.

To estimate pooled median epilepsy prevalence, incidence, mortality and TG we separately fitted random effects models to log-transformed observed estimates using STATA v12 (Stata Corp., TX). LTE and AE were analyzed separately. Similarly, the pooled median NCC prevalence among PWE was obtained. For all meta-analysis we obtained estimates of the median, 95% CI of distribution of true prevalence and incidence by back-transforming the logit scale to the original estimates. The analysis was separately applied to the studies stratified by setting (urban/rural), by age groups (adults/children) and, for NCC prevalence, by diagnostic method (neuroimaging/EITB assay).

Forest plots were used to visualize the heterogeneity among the studies [22]. We used the Cochrane chi-square (χ2) test to examine the null hypothesis that the observed heterogeneity was due to sampling error [23] and calculated the degree of heterogeneity using the statistic I^2^ [24]. A value >50% was considered as substantial heterogeneity.

The influence of NCC prevalence on epilepsy prevalence (LTE and AE), incidence and mortality was assessed using random effects meta-regression. For each country and for each setting (rural/urban), NCC proportion among PWE was considered as a binary variable (low/high) dichotomized according to the median value of the seroprevalence (EITB assay) estimated. We performed both univariate and multivariable analysis adjusting the model for variables that could influence epidemiological estimates: study setting (urban, rural), age of study participants (all, adults, children), method of data collection and ascertainment of epilepsy cases (questionnaires and neurological examination, instrumental ascertainment), type of estimate (point, period), study size (>20,000; 1,000–20,000; <1,000), definition of epilepsy (ILAE 1993, others), screening questionnaire (WHO, others), validation of the questionnaires (validated, not validated) and level of TG dichotomized (≤50%, >50%). Study design (retrospective or prospective) was also a variable evaluated for incidence estimate. Using a backward stepwise procedure, we included in the multivariable model all variables that showed evidence of an association at the significance level p≤0.25 in the univariate analysis. At each step, nonsignificant explanatory variables were removed and only variable with p≤0.05 were retained in the model.

#### Association between CC/NCC and epilepsy

To estimate the association between CC/NCC and epilepsy in LAC we performed a further meta-analysis of case-control studies, applying a random effects model. Odds ratios (ORs) and 95% CI were determined. To account for the different diagnostic methods, the analysis was separately applied to the studies using EITB, and for studies also using neuroimaging.

## Results

### Studies identified

Flowcharts of the literature searches are shown in figure S1 and S2. Of 48 retained articles on epilepsy burden (table S1), 41 reported prevalence, five incidence, 14 TG and five mortality (table S2). Most studies evaluated both adults and children and methods of ascertainment of epilepsy were mainly based on both questionnaire and neurological examination. The screening instruments more frequently adopted were the 1991 WHO questionnaire (WHO, 1991) and the questionnaires used by Placencia [25]–[31] and by Pradilla [32], [33]. The 1993 ILAE definition of AE was the most frequently used but some studies reported a narrower time frame, considering the previous 24 months [33], [34] or the previous 12 months [35].

Thirty-one studies described the proportion of CC/NCC among PWE in LAC (table S3 and S4). Proportion of patients with positive brain CT scan ranged from 8.8% [36] to 70% [37]. Serological diagnosis of CC/NCC with EITB was performed in 16 studies, and the proportion ranged among 0% [38] and 39.5% [30]. Thirteen studies associated to neuroimaging or EITB other ascertainment methods for the diagnosis of CC/NCC, such as serum AbELISA, serum AgELISA, CSF AbELISA, serum ELISA/serum immunofluorescence assay, or CT/CSF test/surgery [39], with proportions ranging from 0% [38] to 41.9% [40].

The association between CC/NCC and epilepsy was evaluated in ten of the 31 studies (table S4). In nine of them the association was significant, with a OR ranging between 2.92 [41] and 12.25 [42]. Only one study reported absence of CC/NCC cases among both PWE and controls [43].

### Meta-analysis

#### Prevalence of epilepsy (LTE and AE) and of NCC among PWE

**Figure 1:**
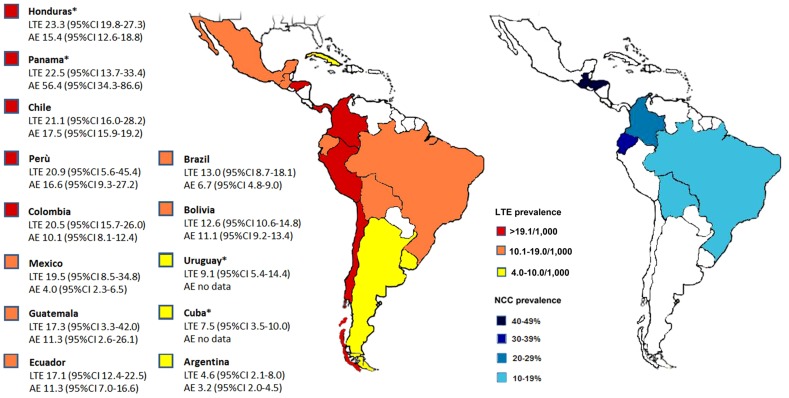
Pooled life-time (LTE), active epilepsy (AE) prevalence (/1,000), NCC prevalence (by CT scan) and 95% confidence intervals in Latin American countries. *estimates obtained from only one study.

**Figure 2:**
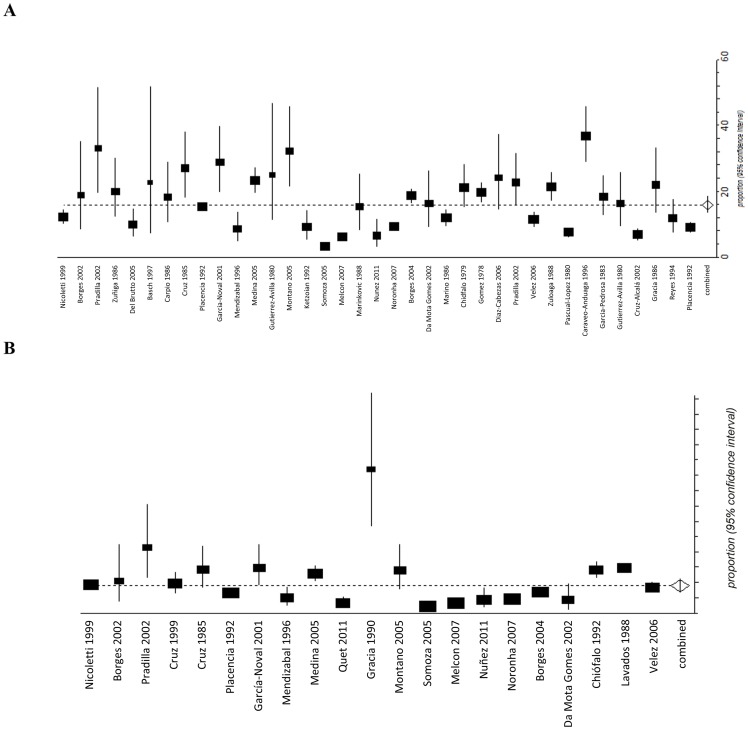
Forest plots for life-time epilepsy prevalence/1,000 (A) and active epilepsy prevalence/1,000 (B) and 95% CI. Pooled LTE prevalence for all studies (N = 37) was 15.8/1,000 (95% CI 13.5–18.3). Pooled AE prevalence for all studies (N = 21) was 10.7/1,000 (95% CI 8.4–13.2).

The estimated median LTE prevalence for all studies combined was 15.8/1,000 (95% CI 13.5–18.3) while median AE prevalence was 10.7/1,000 (95% CI 8.4–13.2) (table 1). In rural areas, LTE prevalence was 18.6/1,000 (95% CI 15.3–22.1) and AE was 13.5/1,000 (95% CI 10.2–17.2), while in urban areas LTE was 14.0/1,000 (95% CI 11.3–17.0) and AE was 7.8/1,000 (95% CI 4.9–11.4). Results of the meta-analysis set by country are illustrated in figure 1. Honduras presented the highest LTE prevalence (23.3/1,000; 95% CI 19.8–27.3) while Argentina the lowest (4.6/1,000; 95% CI 2.1–8.0). The marked variability among studies was attributable to between-study heterogeneity for both LTE (I^2^ = 95.9%; p<0.0001) and AE estimates (I^2^ = 90.4%; p<0.0001) (figure 2). Moreover, significant heterogeneity was evidenced when stratifying on study setting (rural and urban; I^2^>90) and on age group (children and adults; I^2^>90).

**Table 1:** Median lifetime epilepsy prevalence, active epilepsy prevalence, incidence, mortality, treatment gap and neurocysticercosis prevalence among people with epilepsy.

Estimate	Covariate	Number of studies	Median estimate	95% Confidence Intervals
**LTE**	Rural	15	18.6/1,000	15.3–22.1
	Urban	22	14.0/1,000	11.3–17.0
	Total	36	15.8/1,000	13.5–18.3
	Adults	2	33.0/1,000	26.4–41.8
	Children	8	12.3/1,000	7.4–18.3
	All age groups	25	15.7/1,000	13.4–18.1
**AE**	Rural	12*	13.5/1,000	10.2–17.2
	Urban	10*	7.8/1,000	4.9–11.4
	Total	21	10.7/1,000	8.4–13.2
	Adults	2	34.3/1,000	6.6–82.6
	Children	2	3.6/1,000	1.4–6.9
	All age groups	18	10.4/1,000	8.2–12.9
**Incidence**	Rural	3*	138.4/100,000	50.6–269.3
	Urban	3*	121.7/100,000	77.5–175.7
	Total	5	138.2/100,000	83.6–206.4
**Mortality (SMR)**	Rural	1	1.3	0.7–2.39
	Urban	2	/	/
	Total	3	1.4	0.01–6.1
**TG**	Rural	9*	77.8/100	67.4–86.8
	Urban	6*	26.2/100	10.2–46.4
	Total	14	60.6/100	45.3–74.9
**NCC prevalence:** EITB assay	Rural	11	23.7/100	17.6–30.3
	Urban	5	12.1/100	9.3–15.3
	Total	16	19.6/100	14.8–24.9
**NCC prevalence:** CT scan	Rural	9	37.5/100	26.3–49.4
	Urban	11	29.4/100	21.4–37.4
	Total	20	32.3/100	26.0–39.0

AE: active epilepsy; EITB: enzyme-linked immunoelectrotransfer blot; CT scan: computed tomography scan; LTE: lifetime epilepsy; NCC: neurocysticercosis;

SMR: standardized mortality ratio; TG: treatment gap.

* one study (Placencia et al., 1992) reported separated data for both urban and rural settings.

The estimated median NCC proportion among PWE for all LAC was 32.3% (95% CI 26.0–39.0) by CT scan and 19.6% (95% CI 14.8–24.9) by EITB assay (table 1). In rural areas proportion was higher: 37.5% (CT scan) and 23.7% (EITB assay) versus 29.4% (CT scan) and 12.1% (EITB assay) reported in urban areas. Results of the meta-analysis set by country are illustrated in figure 1 and detailed in table 2.

**Table 2:** Neurocysticercosis prevalence among people with epilepsy in Latina American Countries.

		RURAL			URBAN		
Country	Diagnostic method	Number of studies	Median estimate	95% Confidence Intervals	Number of studies	Median estimate	95% Confidence Intervals
**Brazil**	EITB assay	/	/	/	2	11.4/100	5.5–19.1
	CT scan	/	/	/	6	28.2/100	16.9–41.0
**Bolivia**	EITB assay	1	18.8/100	12.5–27.1	/	/	/
	CT scan	1	21.0/100	14.2–29.8	/	/	/
	Del Brutto	1	27.4/100	20.3–35.9	/	/	/
**Colombia**	EITB assay	/	/	/	1	9.8/100	/
	CT scan	/	/	/	1	13.9/100	11.3–17.1
**Ecuador**	EITB assay	2	24.4/100	13.5–37.3	/	/	/
	CT scan	2	40.9/100	17.9–66.3	/	/	/
**Guatemala**	EITB assay	1	17.5/100	10.6–27.4	/	/	/
	CT scan	1	47.4/100	36.5–58.4	/	/	/
**Honduras**	EITB assay	/	/	/	/	/	/
	CT scan	1	13.9/100	5.6–29.1	/	/	/
	Del Brutto	1	36.7/100	27.4–47.0	/	/	/
**Mexico**	EITB assay	2	24.9/100	17.4-33-3	/	/	/
	CT scan	1	70.0/100	39.2–89.7	3	32.1/100	17.1–49.3
**Nicaragua**	EITB assay	/	/	/	1	14.8/100	8.7–23.8
	CT scan	/	/	/	/	/	/
**Panama**	EITB assay	1	0.0/100	/	/	/	/
	CT scan	/	/	/	/	/	/
**Peru**	EITB assay	4	35.6/100	27.1–44.7	1	11.6/100	7.8–17.1
	CT scan	3	40.5/100	28.3–53.3	1	54.1/100	38.4–69.0

EITB: enzyme-linked immunoelectrotransfer blot; CT scan: computed tomography scan.

Multivariable random effects meta-regression (table 3 and 4) showed that higher TG and NCC proportions were significantly associated with higher LTE (OR 3.4; 95% CI 1.6–5.2) and AE (OR 2.7; 95% CI 2.3–5.6) prevalence estimates. Together with study setting and study size, these variables accounted for 61.8% of the observed LTE prevalence heterogeneity. Considering AE prevalence, TG, NCC prevalence , study setting, epilepsy ascertainment and epilepsy definition accounted for 85.4% of the observed heterogeneity.

**Table 3:** Meta-regression of lifetime epilepsy prevalence: Univariate and multivariable analysis.

	UNIVARIATE				MULTIVARIABLE	
	Odds ratio (95% CI)	p- value	Heterogeneity (τ^2^)	Heterogeneity (%)	Odds ratio (95% CI)	p- value
**Study setting**						
Urban	1.0	/			1.0	/
Rural	1.6 (1.1–3.2)	**0.05**	0.48	29.2	1.3 (1.0–2.6)	0.07
**Age group**						
All	1.0	/			1.0	/
Adults	2.3 (1.2–4.1)	**0.03**	0.44	12.9	1.6 (0.9–4.2)	0.5
Children	0.3 (0.2–1.9)	0.09			0.2 (0.1–1.2)	0.6
**Epilepsy ascertainment**						
Q+E	1.0	/			1.0	/
Q+E+T	1.7 (0.6–7.2)	0.1	0.46	7.6	1.1 (0.5–6.3)	0.5
**Type of estimate**						
Point	1.0	/			/	/
Period	0.2 (0.1–5.8)	0.8	0.52	−3.7	/	/
**Study size**						
>20,000	1.0	/			1.0	/
1,000–20,000	1.9 (1.5–3.2)	**0.001**	0.34	32.7	1.4 (1.2–3.1)	**0.04**
<1,000	3.8 (2.8–9.3)	**0.03**			1.8 (1.5–8.2)	**0.05**
**Definition of epilepsy**						
ILAE 1993	1.0	/			/	/
Other definition	0.5 (0.2–3.2)	0.6	0.51	−2.7	/	/
**Questionnaire**						
WHO	1.0	/			/	/
Others	0.2 (0.1–2.4)	0.8	0.52	−3.5	/	/
**Validated questionnaire**						
Yes	1.0	/			/	/
No	0.2 (0.1–9.5)	0.8	0.50	−3.3	/	/
**Treatment gap**						
≤50%	1.0	/			1.0	/
>50%	2.3 (1.3–7.4)	**0.04**	0.26	36.9	2.7 (2.1–6.9)	**0.02**
**NCC prevalence**						
≤median prevalence*	1.0	/			1.0	/
>median prevalence*	1.5 (0.9–5.8)	0.1	0.32	6.7	3.4 (1.3–5.2)	**0.03**

CC: cysticercosis; CI: confidence interval; E: neurological evaluation; NCC: neurocysticercosis Q: questionnaire; T: tool.

* median prevalence (EITB assay) estimates among people with epilepsy in Latin American countries: 12.1% for studies performed in urban areas, 23.7% for studies performed in rural areas.

**Table 4:** Meta-regression of active epilepsy prevalence: Univariate and multivariable analysis.

	UNIVARIATE				MULTIVARIABLE	
	Odds ratio (95% CI)	p- value	Heterogeneity (τ^2^)	Heterogeneity (%)	Odds ratio (95% CI)	p- value
**Study setting**						
Urban	1.0	/			1.0	/
Rural	1.4 (1.1–3.4)	**0.05**	0.30	20.7	1.4 (0.9–3.0)	0.08
**Age group**						
All	1.0	/			/	/
Adults	1.1 (0.7–2.4)	0.9	0.33	−9.7	/	/
Children	1.3 (0.3–1.2)	0.8			/	/
**Epilepsy ascertainment**						
Q+E	1.0	/			1.0	/
Q+E+T	2.0 (1.3–5.7)	**0.05**	0.21	41.8	1.8 (0.8–5.2)	0.09
**Type of estimate**						
Point	1.0	/			/	/
Period	0.2 (0.1–2.3)	0.8	0.32	−8.1	/	/
**Study size**						
>20,000	1.0	/			1.0	/
1,000–20,000	1.2 (1.1–2.0)	**0.05**	0.30	10.7	1.5 (0.8–2.1)	0.1
<1,000	1.6 (0.9–5.3)	0.06			1.7 (0.7–4.2)	0.1
**Definition of epilepsy**						
ILAE 1993	1.0	/			1.0	/
Other definition	0.5 (0.4–1.1)	0.2	0.26	11.3	0.8 (0.2–0.9)	**0.04**
**Questionnaire**						
WHO	1.0	/			/	/
Others	0.5 (0.2–2.5)	0.6	0.33	−10.7	/	/
**Validated questionnaire**						
Yes	1.0	/			/	/
No	1.1 (0.8–7.3)	0.3	0.29	1.4	/	/
**Treatment gap**						
≤50%	1.0	/			1.0	/
>50%	1.7 (1.2–4.5)	**0.05**	0.11	41.6	1.6 (0.8–4.2)	0.09
**NCC prevalence**						
≤median prevalence*	1.0	/			1.0	/
>median prevalence*	2.1 (1.9–6.3)	**0.05**	0.10	12.5	2.7 (2.3–5.6)	**0.05**

CC: cysticercosis; CI: confidence interval; E: neurological evaluation; NCC: neurocysticercosis Q: questionnaire; T: tool.

* median seroprevalence (EITB) estimates among people with epilepsy in Latin American countries: 12.1% for studies performed in urban areas, 23.7% for studies performed in rural areas.

#### Incidence of epilepsy

The estimated median incidence of epilepsy for all the studies combined was 138.2/100,000 persons/year (95% CI 83.6–206.4). In rural settings (table 1), the median incidence rate was 138.4/100,000 persons/year (95% CI 50.6–269.3), higher than that observed in urban settings: 121.7/100,000 persons/year (95% CI 77.5–175.7). The variability among studies was attributable to a between-study heterogeneity (I^2^ = 66.4%; p<0.02). Both the univariate and multivariable models found no consistent associations between incidence estimates, NCC prevalence and TG (table S5).

#### Estimates of mortality and heterogeneity among studies

The study conducted by da Mota Gomes et al. [44] in Brazil was excluded from the meta-analysis because did not provide the denominator (number of PWE), while the study conducted by Devilat Barros et al. [45] was excluded because it considered only children. The overall estimate of SMR (table 1) for all the studies included was 1.4 (95% CI 0.01–6.1). The evaluation of between-study heterogeneity was not significant (I^2^ = 0.0%; p = 0.9).

#### Magnitude of the TG

The overall estimated TG was 60.6% (95% CI 45.3–74.9), clearly different between rural (77.8%; 95% CI 67.4–86.8) and urban (26.2%; 95% CI 10.2–46.4) settings (table 1). There was a wide variability in the TG estimates among studies, attributable to a between-study heterogeneity (I^2^ = 95.0%; p<0.0001). Studies performed in rural settings were associated with higher prevalence than those conduced in urban areas (OR 4.0; 95% CI 2.4–5.2). In the multivariable random effects meta-regression model, study setting was significantly associated with TG and accounted for about 60% of the observed heterogeneity (table S6).

#### Association between CC/NCC and epilepsy

**Figure 3:**
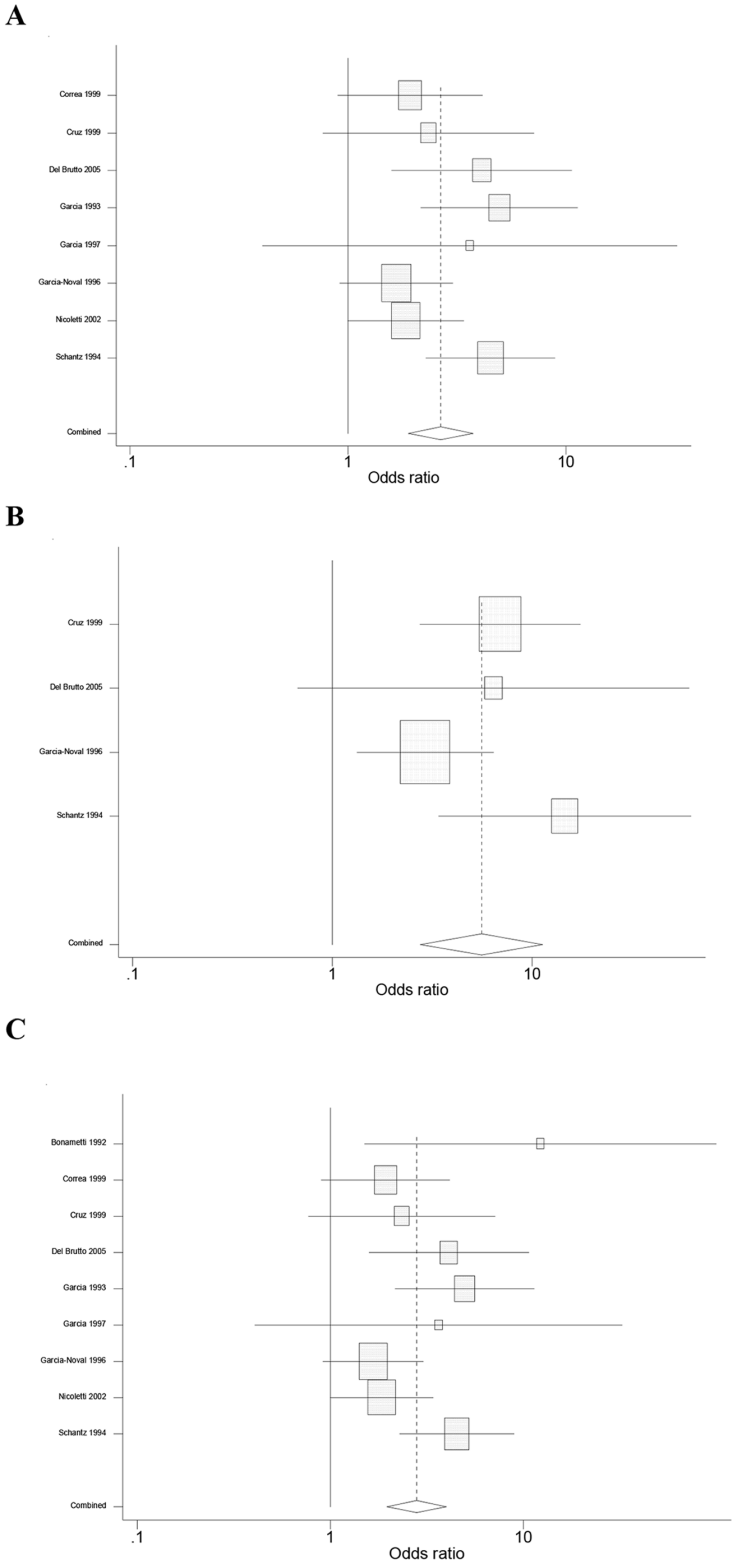
Association between cysticercosis and epilepsy in Latin American Countries. A. Random-effects meta-analysis restricted to studies using EITB (N = 8): common odds ratio (OR) 2.7 (p<0.001). B. Random-effects meta-analysis restricted to studies using brain CT scan (N = 4): common OR 5.6 (p<0.001). C. Random-effects meta-analysis of all the studies (N = 9): common OR (2.8; p<0.001).

One study [38], reporting absence of CC/NCC cases among both PWE and PWOE, was excluded from the meta-analysis. A significant (p<0.001) common OR of 2.8 (95% CI 1.9–4.0) was estimated for all nine studies identified (figure 3). The test of heterogeneity was not significant (p = 0.09), indicating homogeneity of the studies included. The analysis with the eight studies using EITB demonstrated a common OR of 2.7 (95% CI 1.9–3.7; p<0.001). The test for heterogeneity was also not significant (p = 0.06). The meta-analysis of the four studies using neuroimaging yielded an OR of 5.6 (95% CI 2.7–11.3, p<0.001) with non-significant heterogeneity (p = 0.2).

## Discussion

Published data on epilepsy in LAC demonstrated a median LTE prevalence of 15.8/1,000, a median AE prevalence of 10.7/1,000 (both higher in rural areas), a median incidence of epilepsy of 138.2/100,000 and an enormous TG in rural areas. Furthermore a median NCC proportion among PWE of 32.3% (by CT scan) and a consistent association between NCC and epilepsy were found.

To minimize biases we performed a comprehensive systematic review, with particular attention to the main Latin American and Caribbean biomedical databases. Nevertheless, lack of epidemiological data on epilepsy from Nicaragua, Venezuela, Paraguay, Suriname, Guyana and a high information gap on incidence, mortality, TG and NCC prevalence in all LAC were pointed out, confirming epilepsy and NCC as major neglected diseases in this region.

The elevated burden of these diseases should be regarded as a primary public-health issue in LAC, especially in rural settings. Although slightly lower, our estimates were close to those reported in a previous meta-analysis (LTE prevalence 17.8/1,000; AE prevalence 12.4/1,000, [7]) and above the median values reported in a recent work considering both developed and developing countries [3]. Also epilepsy incidence in urban and rural setting was greater than that reported in another study analysing low- and middle-income countries [6]. The pooled NCC proportion among PWE was 32.3% (95% CI 26.0–39.0), higher than 29.0% (95% CI 22.9–35.5) reported in a meta-analysis including both rural and urban areas worldwide [12]. NCC proportion found among PWE in rural LAC (37.5%) appeared little lower than that reported in studies applying same criteria in rural Africa (Burkina Faso 46.9%) [46], rural India (40%) [47] and higher than that reported in rural Tanzania (17.9%) [48]. Considering urban areas, our estimate (29.4%) was similar to that reported in South Africa (28.0%) [49] and in urban India (28.4%) [50].

For the first time, to the best of our knowledge, this study demonstrated the influence of NCC prevalence on LTE and AE prevalence in LAC: countries with a NCC proportion (EITB assay) higher than 12.1%, among PWE living in urban areas, and than 23.7%, among PWE living in rural areas, presented higher LTE and AE estimates. We are aware that seropositivity at the EITB reflects an exposure to the parasite not necessary accompanied by a CNS involvement. However, this assay presents a high sensitivity and specificity and, together with the presence of compatible clinical manifestations (such as epilepsy) in people living in endemic areas, allows to formulate the diagnosis of probable NCC according the worldwide accepted diagnostic criteria for NCC [17]. We could then state that NCC prevalence seems to affect both LTE and AE prevalence, and that it could be considered a source of variability of prevalence estimates across LAC. The association between CC/NCC and epilepsy in LAC was evaluated in nine studies using “prevalent” cases. There was a consistent and significant association between epilepsy and CC/NCC, with ORs of 2.7 for studies using EITB serology and 5.6 for studies performing brain CT scan. Studies associating brain imaging to serology are likely more accurate as include those cases who present calcified or single parenchymal cyst which may be asymptomatic or seronegative [51]. Previous meta-analysis data from LAC did not exist, while in Africa a 3.4 to 3.8-fold increased risk for developing epilepsy was reported [52].

The meta-regression analysis has also pointed out an influence of TG on both AE and LTE estimates: since countries with higher TG are those presenting a larger burden of untreated epilepsy, the consequent increased number of active cases could lead to higher AE prevalence. Moreover, as AE prevalence is included in LTE measures (about the 38% for rural and 59% in urban population of developing countries; [3]) the role of TG could also be reflected on LTE estimates. On the other hand, in countries with lower TG, “not-active” cases are probably more frequent and more difficult to detect in prevalence surveys, leading to an underestimation of the LTE prevalence.

When considered together, TG and NCC prevalence together explained a very great amount of AE prevalence variability (up to 85.4%) suggesting that interventions on these modifiable factors could result in important reduction of AE burden.

Finally, our data showed a non significant increase in mortality in PWE in LAC (SMR 1.4; 95% CI 0.01–6.1). Studies in developed countries have reported mortality rates two to three times higher in PWE than in the general population [53]. This increase includes direct and indirect consequences of epilepsy, as well as underlying disorders responsible for secondary epilepsies [54].We found only five eligible papers reporting SMR in LAC (Argentina, Bolivia, Brazil, Chile and Ecuador) ranging from 0.76 in Brazil [44] to 6.3 in Ecuador [55]. These studies presented a prevalent cohort designs that might have underestimate short-term mortality, as patients with more serious disease die earlier and are not included in the observation period. Furthermore, SMR is highest in symptomatic epilepsy (ranging from 2.2–6.5, [53]) while, in idiopathic epilepsy, conflicting results have been reported, often showing a non significant increase, as the one found here. Only in the Bolivian study [56], SMR was estimated stratifying by symptomatic and idiopathic epilepsy, with increased mortality reported only among patients with symptomatic epilepsy (SMR = 3.0; 95% CI 1.2–6.3).

Concluding, this systematic review demonstrated a high burden of epilepsy and of NCC in LAC with marked detriment of rural areas, identified two important modifiable factors related to epilepsy prevalence and a consistent association between NCC and epilepsy in LAC. We are aware about the possible loss of power related to the dichotomization of continuous variables in the meta-regression. However, the paucity of studies found on NCC and on TG in particular, made our analysis not able to support a larger number of categories. Moreover, the reported narrow CIs suggest that the loss of statistical power was minimal. Additional data are needed to better understand the possible sources of heterogeneity among countries and determine the situation in those regions that are still under the shadows.

## Supporting Information

Checklist S1 PRISMA checklist.
(DOCX)

Figure S1 PRISMA flow chart of the literature search on epilepsy burden (prevalence, incidence, mortality, treatment gap) in Latin America.
(DOCX)

Figure S2 PRISMA flow chart of the literature search on cysticercosis and epilepsy in Latin America.
(DOCX)

Table S1 Prevalence, incidence, treatment gap of epilepsy and characteristics of the included studies.
(DOC)

Table S2 Mortality of epilepsy from the included.
(DOC)

Table S3 Study Description of cysticerosis (CC) or neurocysticercosis (NCC) in Patients With Epilepsy (without control group).
(DOC)

Table S4 Studies on the association between cysticercosis/neurocysticerosis and epilepsy in Latin American Countries.
(DOC)

Table S5 Meta-regression of epilepsy incidence: univariate and multivariable analysis.
(DOC)

Table S6 Meta-regression of epilepsy treatment gap (TG): univariate and multivariable analysis.
(DOC)

## References

[1] Scott RA, Lhatoo SD, Sander JW (2001) The treatment of epilepsy in developing countries: where do we go from here? Bull World Health Organ 79: 344–351. 11357214 PMC2566404

[2] de Boer HM, Mula M, Sander JW (2008) The global burden and stigma of epilepsy. Epilepsy Behav 12: 540–546. 18280210 10.1016/j.yebeh.2007.12.019

[3] Ngugi AK, Bottomley C, Kleinschmidt I, Sander JW, Newton CR (2010) Estimation of the burden of active and life-time epilepsy: a meta-analytic approach. Epilepsia 51: 883–890. 20067507 10.1111/j.1528-1167.2009.02481.x PMC3410521

[4] Declaration of Santiago on epilepsy in Latin America (2002) Epilepsia 43: 42. 12190980 10.1046/j.1528-1157.43.s6.2.x

[5] Yemadje LP, Houinato D, Quet F, Druet-Cabanac M, Preux PM (2011) Understanding the differences in prevalence of epilepsy in tropical regions. Epilepsia 52: 1376–1381. 21627649 10.1111/j.1528-1167.2011.03099.x

[6] Ngugi AK, Kariuki SM, Bottomley C, Kleinschmidt I, Sander JW, et al (2011) Incidence of epilepsy: a systematic review and meta-analysis. Neurology 77: 1005–1012. 21893672 10.1212/WNL.0b013e31822cfc90 PMC3171955

[7] Burneo JG, Tellez-Zenteno J, Wiebe S (2005) Understanding the burden of epilepsy in Latin America: a systematic review of its prevalence and incidence. Epilepsy Res 66: 63–74. 16125900 10.1016/j.eplepsyres.2005.07.002

[8] Leonardi M, Ustun TB (2002) The global burden of epilepsy. Epilepsia 43 Suppl 6: 21–25. 10.1046/j.1528-1157.43.s.6.11.x 12190974

[9] Brooker S, Alexander N, Geiger S, Moyeed RA, Stander J, et al (2006) Contrasting patterns in the small-scale heterogeneity of human helminth infections in urban and rural environments in Brazil. Int J Parasitol 36: 1143–1151. 16814294 10.1016/j.ijpara.2006.05.009 PMC1783908

[10] Preux PM, Druet-Cabanac M (2005) Epidemiology and aetiology of epilepsy in sub-Saharan Africa. Lancet Neurol 4: 21–31. 15620854 10.1016/S1474-4422(04)00963-9

[11] Del Brutto OH (2012) Neurocysticercosis: a review. ScientificWorldJournal 2012: 159821. 22312322 10.1100/2012/159821 PMC3261519

[12] Ndimubanzi PC, Carabin H, Budke CM, Nguyen H, Qian YJ, et al (2010) A systematic review of the frequency of neurocyticercosis with a focus on people with epilepsy. PLoS Negl Trop Dis Nov 2;4: e870. 10.1371/journal.pntd.0000870 PMC2970544 21072231

[13] Savioli LS, Daumerie D (2010) First WHO report on neglected tropical diseases: working to overcome the global impact of neglected tropical diseases. Geneva: World Health Organisation;1–169.

[14] Coyle CM, Mahanty S, Zunt JR, Wallin MT, Cantey PT, et al (2012) Neurocysticercosis: neglected but not forgotten. PLoS Negl Trop Dis 6: e1500. 22666505 10.1371/journal.pntd.0001500 PMC3362619

[15] ILAE (1993) Guidelines for epidemiologic studies on epilepsy. Commission on Epidemiology and Prognosis, International League Against Epilepsy. Epilepsia Jul–Aug;34: 592–596. 10.1111/j.1528-1157.1993.tb00433.x 8330566

[16] Meinardi H, Scott RA, Reis R, Sander JW (2001) The treatment gap in epilepsy: the current situation and ways forward. Epilepsia 42: 136–149. 11207798 10.1046/j.1528-1157.2001.32800.x

[17] Del Brutto OH, Rajshekhar V, White AC Jr, Tsang VC, Nash TE, et al (2001) Proposed diagnostic criteria for neurocysticercosis. Neurology 57: 177–183. 11480424 10.1212/wnl.57.2.177 PMC2912527

[18] Garcia HH, Martinez M, Gilman R, Herrera G, Tsang VC, et al (1991) Diagnosis of cysticercosis in endemic regions. The Cysticercosis Working Group in Peru. Lancet 338: 549–551. 1678809 PMC2913119

[19] Rosas N, Sotelo J, Nieto D (1986) ELISA in the diagnosis of neurocysticercosis. Arch Neurol Apr;43: 353–356. 10.1001/archneur.1986.00520040039016 3954618

[20] Ramos-Kuri M, Montoya RM, Padilla A, Govezensky T, Díaz ML, et al (1992) Immunodiagnosis of neurocysticercosis. Disappointing performance of serology (enzymelinked immunosorbent assay) in an unbiased sample of neurological patients. Arch Neurol 49: 633–636. 1596199 10.1001/archneur.1992.00530300069012

[21] Gabriël S, Blocher J, Dorny P, Abatih EN, Schmutzhard E, et al (2012) Added value of antigen ELISA in the diagnosis of neurocysticercosis in resource poor settings. PLoS Negl Trop Dis 6 ((10)): e1851. 23094118 10.1371/journal.pntd.0001851 PMC3475663

[22] Lewis S, Clarke M (2001) Forest plots: trying to see the wood and the trees. BMJ 322: 1479–1480. 11408310 10.1136/bmj.322.7300.1479 PMC1120528

[23] Higgins JP, Thompson SG (2002) Quantifying heterogeneity in a meta-analysis. Stat Med 21: 1539–1558. 12111919 10.1002/sim.1186

[24] Higgins JP, Thompson SG, Deeks JJ, Altman DG (2003) Measuring inconsistency in meta-analyses. BMJ 327: 557–560. 12958120 10.1136/bmj.327.7414.557 PMC192859

[25] Placencia M, Sander JW, Shorvon SD, Ellison RH, Cascante SM (1992) Validation of a screening questionnaire for the detection of epileptic seizures in epidemiological studies. Brain 115 ((Pt 3)) 783–794. 1628202 10.1093/brain/115.3.783

[26] Caraveo-Andagua J, Medina-Mora ME, Rascon ML, Villatoro J, Martinez-Velez A, et al (1996) La prevalencia de los trastornos psiquiatricos en la poblacion urbana adulta en México. Salud Mental 19: 14–21.

[27] Cruz ME, Schantz PM, Cruz I, Espinosa P, Preux PM, et al (1999) Epilepsy and neurocysticercosis in an Andean community. Int J Epidemiol 28: 799–803. 10480714 10.1093/ije/28.4.799

[28] Del Brutto OH, Santibanez R, Idrovo L, Rodriguez S, Diaz-Calderon E, et al (2005) Epilepsy and neurocysticercosis in Atahualpa: a door-to-door survey in rural coastal Ecuador. Epilepsia 46: 583–587. 15816956 10.1111/j.0013-9580.2005.36504.x

[29] Medina MT, Duron RM, Martinez L, Osorio JR, Estrada AL, et al (2005) Prevalence, incidence, and etiology of epilepsies in rural Honduras: the Salama Study. Epilepsia 46: 124–131. 15660778 10.1111/j.0013-9580.2005.11704.x

[30] Montano SM, Villaran MV, Ylquimiche L, Figueroa JJ, Rodriguez S, et al (2005) Neurocysticercosis: association between seizures, serology, and brain CT in rural Peru. Neurology 65: 229–233. 16043791 10.1212/01.wnl.0000168828.83461.09

[31] Diaz-Cabezas R, Ruano-Restrepo MI, Chacon-Cardona JA, Vera-Gonzalez A (2006) [Neuroepidemiology profile of the central zone of the department of Caldas (Colombia), years 2004–2005]. Rev Neurol 43: 646–652. 17133324

[32] Pradilla G, Vesga BE, Leon-Sarmiento FE, Bautista LE, Nunez LC, et al (2002) [Neuroepidemiology in the eastern region of Colombia]. Rev Neurol 34: 1035–1043. 12134301

[33] Borges MA, Barros EP, Zanetta DM, Borges AP (2002) [Prevalence of epilepsy in Bakairi indians from Mato Grosso State, Brazil]. Arq Neuropsiquiatr 60: 80–85. 11965413 10.1590/s0004-282x2002000100014

[34] Noronha AL, Borges MA, Marques LH, Zanetta DM, Fernandes PT, et al (2007) Prevalence and pattern of epilepsy treatment in different socioeconomic classes in Brazil. Epilepsia 48: 880–885. 17326788 10.1111/j.1528-1167.2006.00974.x

[35] Placencia M, Shorvon SD, Paredes V, Bimos C, Sander JW, et al (1992) Epileptic seizures in an Andean region of Ecuador. Incidence and prevalence and regional variation. Brain 115 ((Pt 3)) 771–782. 1628201 10.1093/brain/115.3.771

[36] Valenca MM, Valenca LP (2000) [Etiology of the epileptic seizures in Recife city, Brazil: study of 249 patients]. Arq Neuropsiquiatr 58: 1064–1072. 11105074 10.1590/s0004-282x2000000600014

[37] Schantz PM, Sarti E, Plancarte A, Wilson M, Criales JL, et al (1994) Community-based epidemiological investigations of cysticercosis due to Taenia solium: comparison of serological screening tests and clinical findings in two populations in Mexico. Clin Infect Dis 18: 879–885. 8086547 10.1093/clinids/18.6.879

[38] Gracia F, Chavarria R, Archbold C, Larreategui M, Castillo L, et al (1990) Neurocysticercosis in Panama: preliminary epidemiologic study in the Azuero region. Am J Trop Med Hyg 42: 67–69. 2301707 10.4269/ajtmh.1990.42.67

[39] Arruda W, Camargo N, Coelho R (1990) Neurocysticercosis: an epidemiological survey in two small rural communities. Arq Neuropsiquiatr 48: 419–424. 2094187 10.1590/s0004-282x1990000400004

[40] Silva-Vergara ML, Vieira C, Castro JH, Micheletti LG, Otaño AS, et al (1994) [Neurologic and laboratory findings in a population of an endemic area for taeniasis-cysticercosis, Lagamar, MG, Brazil (1992–1993)]. Rev Inst Med Trop Sao Paulo 36: 335–342. 7732264 10.1590/s0036-46651994000400006

[41] Garcia-Noval J, Allan JC, Fletes C, Moreno E, DeMata F, et al (1996) Epidemiology of Taenia solium taeniasis and cysticercosis in two rural Guatemalan communities. Am J Trop Med Hyg 55: 282–289. 8842116 10.4269/ajtmh.1996.55.282

[42] Bonametti AM, Basile MA, Vaz AJ, Baldy JL, Takiguti CK (1992) [The positivity index of the immunoenzyme reaction (ELISA) for cysticercosis in the cerebrospinal fluid (CSF) and in the serum of epilepsy patients]. Rev Inst Med Trop Sao Paulo 34: 451–458. 1342110

[43] Gracia F, de Lao SL, Castillo L, Larreategui M, Archbold C, et al (1990) Epidemiology of epilepsy in Guaymi Indians from Bocas del Toro Province, Republic of Panama. Epilepsia 31: 718–723. 2245802 10.1111/j.1528-1157.1990.tb05512.x

[44] da Mota Gomes M (2011) Mortality from epilepsy Brazil (capitals), 1980–2007. Arq Neuropsiquiatr 69: 166–169. 21537553 10.1590/s0004-282x2011000200004

[45] Devilat Barros M, Rivera Gómez G, Gómez Muñoz V, Sepulveda Olmos JP (2004) [Mortality in children with epilepsy. A clinical prospective study]. Rev Neurol Apr 1–15 ;38: 607–614. 15098179

[46] Millogo A, Nitiéma P, Carabin H, Boncoeur-Martel MP, Rajshekhar V, Tarnagda Z, Praet N, et al (2012) Prevalence of neurocysticercosis among people with epilepsy in rural areas of Burkina Faso. Epilepsia Dec;53 ((12)): 2194–202. 10.1111/j.1528-1167.2012.03687.x PMC5459323 23148555

[47] Raina SK, Razdan S, Pandita KK, Sharma R, Gupta VP, et al (2012) Active epilepsy as indicator of neurocysticercosis in rural northwest India. Epilepsy Res Treat 2012: 802747. 22957243 10.1155/2012/802747 PMC3420514

[48] Winkler AS, Blocher J, Auer H, Gotwald T, Matuja W, et al (2009) Epilepsy and neurocysticercosis in rural Tanzania-An imaging study. Epilepsia 50: 987–993. 19054402 10.1111/j.1528-1167.2008.01867.x

[49] van As AD, Joubert J (1991) Neurocysticercosis in 578 black epileptic patients. S Afr Med J Oct 5; 80 ((7)): 327–8. 1925838

[50] Rajshekhar V, Raghava MV, Prabhakaran V, Oommen A, Muliyil J (2006) Active epilepsy as an index of burden of neurocysticercosis in Vellore district, India. Neurology Dec 26;67 ((12)): 2135–9. 10.1212/01.wnl.0000249113.11824.64 17190933

[51] Prabhakaran V, Rajshekhar V, Murrell KD, Oommen A (2004) Taenia solium metacestode glycoproteins as diagnostic antigens for solitary cysticercus granuloma in Indian patients. Trans R Soc Trop Med Hyg 98: 478–484. 15186936 10.1016/j.trstmh.2003.12.006

[52] Quet F, Guerchet M, Pion SD, Ngoungou EB, Nicoletti A, et al (2010) Meta-analysis of the association between cysticercosis and epilepsy in Africa. Epilepsia 51: 830–837. 19919664 10.1111/j.1528-1167.2009.02401.x

[53] Forsgren L, Hauser WA, Olafsson E, Sander JW, Sillanpaa M, et al (2005) Mortality of epilepsy in developed countries: a review. Epilepsia 46 Suppl 11: 18–27. 16393174 10.1111/j.1528-1167.2005.00403.x

[54] Beghi E, Leone M, Solari A (2005) Mortality in patients with a first unprovoked seizure. Epilepsia 46 Suppl 11: 40–42. 16393178 10.1111/j.1528-1167.2005.00407.x

[55] Carpio A, Bharucha NE, Jallon P, Beghi E, Campostrini R, et al (2005) Mortality of epilepsy in developing countries. Epilepsia 46 Suppl 11: 28–32. 16393175 10.1111/j.1528-1167.2005.00404.x

[56] Nicoletti A, Sofia V, Vitale G, Bonelli SI, Bejarano V, et al (2009) Natural history and mortality of chronic epilepsy in an untreated population of rural Bolivia: a follow-up after 10 years. Epilepsia Oct;50: 2199–2206. 10.1111/j.1528-1167.2009.02174.x 19563350

