# Supplementary material for: Epilepsy and Neurocysticercosis in Latin America: A Systematic Review and Meta-analysis
Source: PLoS Negl Trop Dis. 2013 Oct 31;7(10):e2480. doi: 10.1371/journal.pntd.0002480 (PMC3814340; doi:10.1371/journal.pntd.0002480)
Supplement: Figure S1 — PRISMA flow chart of the literature search on epilepsy burden (prevalence, incidence, mortality, treatment gap) in Latin America. (DOCX) [file pntd.0002480.s002.docx]

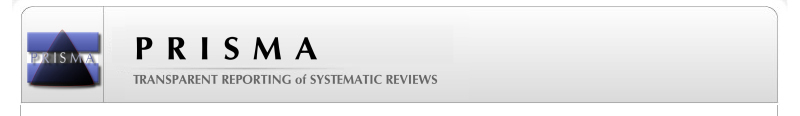
**PRISMA 2009**

**Flow Diagram of the literature search on epilepsy burden (prevalence, incidence, mortality, treatment gap) in Latin America**

## Identification

Records identified through database searching
(n = 2,133)

MEDLINE 1,603

IMBIOMED 134

LILACS 122

EMBASE 52

SciELO 75

PAHO 94

WHOLIS 53

Records excluded
(n = 1,329)

Full-text articles excluded,
(n = 17)

Published as correspondence/letter/editorial= 4

Published as review article= 9

Duplicate studies= 3

Denominator data not provided= 1

Studies included in quantitative synthesis (meta-analysis)
(n = 48)

Studies included in qualitative synthesis
(n = 48)

Full-text articles assessed for eligibility
(n = 65)

Records screened
(n = 1,394)

Records after duplicates removed
(n = 1,394)

Additional records identified through hand-search
(n = 3)

## Eligibility

## Included

## Screening
