# Supplementary material for: Epilepsy and Neurocysticercosis in Latin America: A Systematic Review and Meta-analysis
Source: PLoS Negl Trop Dis. 2013 Oct 31;7(10):e2480. doi: 10.1371/journal.pntd.0002480 (PMC3814340; doi:10.1371/journal.pntd.0002480)
Supplement: Figure S2 — PRISMA flow chart of the literature search on cysticercosis and epilepsy in Latin America. (DOCX) [file pntd.0002480.s003.docx]

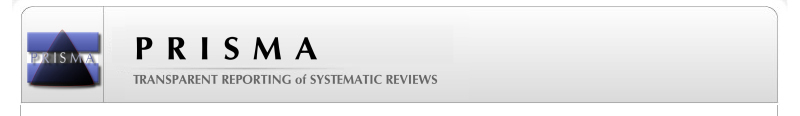
**PRISMA 2009**

**Flow Diagram of the literature search on cysticercosis and epilepsy in Latin America**

## Identification

Records identified through database searching
(n = 889)

MEDLINE 143

IMBIOMED 405

LILACS 87

EMBASE 41

Cochrane Library 12

SciELO 35

PAHO 91

WHOLIS 75

Full-text articles excluded,
(n = 7)

Published as review= 1

Duplicate studies= 2

Number of PWE CC+ not reported= 4

Studies included in quantitative synthesis (meta-analysis)
(n = 9)

Studies included in qualitative synthesis
(n = 31)

Full-text articles assessed for eligibility
(n = 38)

Records excluded
(n = 704)

Records screened
(n = 742)

Records after duplicates removed
(n = 742)

Additional records identified through hand-search
(n = 4)

## Eligibility

## Included

## Screening
