# Supplementary material for: Epilepsy and Neurocysticercosis in Latin America: A Systematic Review and Meta-analysis
Source: PLoS Negl Trop Dis. 2013 Oct 31;7(10):e2480. doi: 10.1371/journal.pntd.0002480 (PMC3814340; doi:10.1371/journal.pntd.0002480)
Supplement: Table S1 — Prevalence, incidence, treatment gap of epilepsy and characteristics of the included studies. (DOC) [file pntd.0002480.s004.doc]

| **Reference** | **Year of study** | **Country** | **Epilepsy definition** | **Methods of ascertainment** | **Questionnaire**  **(performed by)** | **Validation** | **Population**    **N age** | | **Prevalence**  **/1,000**    **LT AE** | | | **Incidence**  **/100.000** | **Treatment**  **gap %** | | **Number**  **of cases (AE)** | |
| --- | --- | --- | --- | --- | --- | --- | --- | --- | --- | --- | --- | --- | --- | --- | --- | --- |
| **RURAL** | | | | | | | | | | | | | | | | |
| Nicoletti et al., 1999 | 1994 | Bolivia | ILAE 1993 | Q, E, T (EEG) | WHO, SNES  (health workers) | pretested | 9,955 | All | point | 12.3 | 11.1 | / | | 90.0 | | 112 |
| Borges et al.,  2002 | 1999 | Brasil | ILAE 1993* | Q, E, T (EEG) | Placencia 1992  (medicine students) | validated | 481 | All | period | 18.6 | 12.4 | / | | 66.6 | | 6 |
| Pradilla et al., 2002 | 1995 | Colombia | ILAE 1993 | Q, E | WHO  (local personnel) | validated | 544 | All | point | 33.0 | 25.7 | / | | / | | / |
| Zuñiga et al.,  1986 | 1984 | Colombia | provided | Q | / | / | 1053 | All | point | 19.9 | / | / | | / | | / |
| Del Brutto et al., 2005 | 2003 | Ecuador | ILAE 1993 | Q, E, T (EEG, TC) | Placencia 1992  (rural doctors) | validated | 2415 | All | point | 9.94 | / | / | | 46 | | 18 |
| Basch et al.,  1997 | 1995 | Ecuador | ILAE 1993 | Q, E | Ad hoc | pretested | 221 | All | point | 22.6 | / | / | | / | | / |
| Carpio et al.,  1986 | 1983 | Ecuador | provided | Q, E | / | / | 935 | All | point | 18.2 | / | / | | / | | / |
| Cruz et al.,  1999 | 1992 | Ecuador | ILAE 1993 | Q, E, T (EEG, TC) | WHO  (paramedical personnel) | validated | 2723 | All | point | / | 11.4 | / | | / | | / |
| Cruz et al.,  1985 | 1982 | Ecuador | provided | Q, E | / | pretested | 1113 | All | point | 27 | 17.1 | / | | / | | / |
| Placencia, et al., 1992 | 1987 | Ecuador | provided* | Q, E | Placencia 1992  (secondary school students) | validated | 59353 | All | point | 15.4 | 8 | 122-190 | | 79 (Placencia et al., 1994) | | 575 |
| Garcia-Noval et al.,2001 | 1992 | Guatemala | provided | Q, E, T (EEG, TC) | Ad hoc  (medical students) | Not validated | 2292 | Adults | period | 28.5 | 18 | / | | / | | / |

**Table S1. Prevalence, Incidence, Treatment gap of epilepsy and characteristics of the included studies (N=41)**

(continued)

**Table S1. Prevalence, Incidence, Treatment gap of epilepsy and characteristics of the included studies (N=41)(continued)**

| **Reference** | **Year of study** | **Country** | **Epilepsy definition** | **Methods of ascertainment** | **Questionnaire**  **(performed by)** | **Validation** | **Population**    **N age** | | **Prevalence/1,000**    **LT AE** | | | **Incidence**  **/100.000** | **Treatment**  **gap %** | | **Number**  **of cases (AE)** |
| --- | --- | --- | --- | --- | --- | --- | --- | --- | --- | --- | --- | --- | --- | --- | --- |
| Mendizabal et al., 1996 | 1991 | Guatemala | provided | Q, E | WHO | / | 1882 | All | point | 8.5 | 5.8 | / | | 68.8 | 11 |
| Medina et al., 2005 | 1997 | Honduras | ILAE 1993 | Q, E, T (EEG, TC) | WHO  (neurologists) | validated | 6473 | All | point | 23.3 | 15.4 | 57 | | 53.3 | 100 |
| Quet et al.,  2011 | 2007 | Mexico | ILAE 1993 | Q, E | WHO  (students) | Not validated | 4008 | All | point | / | 3.9 | / | | 75 | 16 |
| Gutierrez-Avila et al., 1980 | 1980° | Mexico | provided | Q, E, T (EEG) | / | / | 360 | Children | / | 25 | / | / | | / | / |
| Gracia et al.,  1990 | 1988 | Panama | provided | Q, E | / | / | 337 | Adults | / | / | 57 | / | | 100 | 19 |
| Montano et al., 2005 | 2000 | Perù | ILAE 1993 | Q, E, T (TC) | Placencia 1992  (field workers) | validated | 903 | All | period | 32.1 | 16.6 | 162.3  (Villaran) | | 100 | 15 |
| Ketzoian et al., 1992 | 1990 | Uruguay | WHO | Q, E | WHO  (medical students) | tested | 1975 | All | point | 9.1 | / | / | | / | / |
| **URBAN** | | | | | | | | | | | | | | | |
| Somoza et al., 2005 | 1991 | Argentina | ILAE 1993 | Q, E | Ad hoc  (self-administred) | validated | 26270 | Children | point | 3.2 | 2.6 | / | | 7.1 | 68 |
| Melcon et al., 2007 | 1998 | Argentina | ILAE 1993 | Q, E | Copiah country study  (trained personnel) | validated | 17049 | All | point | 6.2 | 3.8 | / | | 22 | 64 |
| Marinkovic et al., 1988 | 1988° | Bolivia | provided | Q, E | / | / | 915 | All | / | 15.3 | / | / | | / | / |
| Nunez et al.  2011 | 2003 | Brasil | ILAE 1993 | Q, E | Fernandes 1992  (trained personnel) | validated | 1687 | Children | point | 6.5 | 5.3 | 177.8 | | / | / |
| Noronha et al., 2007 | 2002 | Brasil | ILAE 1993* | Q, E | Placencia 1992  (health-care personnel) | validated | 96300 | All | period | 9.2 | 5.4 | / | | 38  (Li 07) | 290 |

(continued)

**Table S1. Prevalence, Incidence, Treatment gap of epilepsy and characteristics of the included studies (N=41)(continued)**

| **Reference** | **Year of study** | **Country** | **Epilepsy definition** | **Methods of ascertainment** | **Questionnaire**  **(performed by)** | **Validation** | **Population**  **N age** | | **Prevalence/1,000**    **LT AE** | | | **Incidence**  **/100.000** | **Treatment**  **gap %** | | | **Number**  **of cases (AE)** |
| --- | --- | --- | --- | --- | --- | --- | --- | --- | --- | --- | --- | --- | --- | --- | --- | --- |
| Borges et al.,  2004 | 2001 | Brasil | ILAE 1993 | Q, E | WHO  (trained personnel) | validated | 17293 | All | period | 18.6 | 8.2 | / | | 60 | 141 | |
| Da Mota Gomes et al., 2002 | 2000 | Brasil | ILAE 1993 | Q, E | Placencia 1992  (students) | Not validated | 982 | All | point | 16.3 | 5.1 | / | | 0 | 5 | |
| Marino et al.,1986 | 1984 | Brasil | provided | Q, E | / | / | 7603 | All | / | 11.9 | / | / | | / | / | |
| Chiófalo et al., 1979 | 1975 | Chile | Rose 1973 | Q, E, T (EEG) | Rose 1973  (students, social workers) | Not validated | 2085 | Children | point | 21.1 | / | / | | / | / | |
| Chiófalo et al., 1992 | 1992° | Chile | WHO | Q, E | WHO | / | 7195 | All | point | / | 17 | / | | / | / | |
| Lavados et al., 1992 | 1988 | Chile | provided | Medical records | / | / | 17694 | All | point | / | 17.7 | 113 | | / | / | |
| Gomez et al.,  1978 | 1974 | Colombia | provided | Q, E, T (EEG) | / | / | 8658 | All | point | 19.5 | / | / | | / | / | |
| Diaz-Cabezas et al., 2006 | 2004 | Colombia | ILAE 1993 | Q, E | Pradilla  (students) | validated | 787 | All | point | 24 | / | / | | / | / | |
| Pradilla et al., 2002 | 1983-1992 | Colombia | ILAE 1993 | Q, E | WHO  (health workers) | validated | 1454 | All | period | 22.7 | / | / | | / | / | |
| Velez et al.,  2006 | 1996 | Colombia | ILAE 1993 | Q, E, T (EEG) | WHO | validated | 8910 | All | period | 11.3 | 10.1 | / | | / | / | |
| Zuloaga et al., 1988 | 1983 | Colombia | provided | Q, E | / | / | 4549 | All | point | 21.4 | / | / | | / | / | |
| Pascual et al.,1980 | 1980* | Cuba | provided | Q, E | Home calls | / | 14445 | Children | point | 6 | / | / | | / | / | |

(continued)

**Table S1. Prevalence, Incidence, Treatment gap of epilepsy and characteristics of the included studies (N=41)(continued)**

| **Reference** | **Year of study** | **Country** | **Epilepsy definition** | **Methods of ascertainment** | **Questionnaire**  **(performed by)** | **Validation** | **Population**  **N age** | | **Prevalence/1,000**    **LT AE** | | | **Incidence**  **/100.000** | **Treatment**  **gap %** | | **Number**  **of cases (AE)** | |
| --- | --- | --- | --- | --- | --- | --- | --- | --- | --- | --- | --- | --- | --- | --- | --- | --- |
| Placencia, et al  1992 | 1987 | Ecuador | provided* | Q, E | Placencia 1992  (secondary school students) | validated | 12768 | All | point | 9.1 | 8 | 122-190 | | 79 (Placencia et al., 1994) | | 575 |
| Caraveo-Anduaga et al., 1996 | 1988 | Mexico | WHO | Q | WHO | validated | 1984 | Adults | point | 36.8 | / | / | | / | | / |
| Garcia-Pedrosa et al., 1983 | 1975 | Mexico | provided | Q, E, T (EEG) | / | / | 2027 | Children | / | 18.3 | / | / | | / | | / |
| Gutierrez-Avila et al., 1980 | 1978 | Mexico | provided | Q, E, T (EEG) | / | / | 1042 | Children | / | 16 | / | / | | / | | / |
| Cruz-Alcalà et al.,  1999 | 1999 | Mexico | ILAE 1993 | Q, E | Ad hoc  (students) | Not validated | 9082 | All | period | 6.8 | / | / | | / | | / |
| Gracia et al.,  1986 | 1986 | Panama | provided | Q, E | / | / | 955 | All | / | 22.5 | / | / | | / | | / |
| Reyes et al.,  1994 | 1992 | Perù | provided | Q | / | / | 2016 | Children | / | 11.9 | / | / | | / | | / |

AE: active epilepsy ; E: neurological evaluation ; LTE: lifetime epilepsy ; Q: questionnaire; T: tool (EEG: electroencephalography, CT: brain computed tomography).

°: year of publication.

*: active epilepsy: one episode in the past year or in the past two years.
