# Supplementary material for: Epilepsy and Neurocysticercosis in Latin America: A Systematic Review and Meta-analysis
Source: PLoS Negl Trop Dis. 2013 Oct 31;7(10):e2480. doi: 10.1371/journal.pntd.0002480 (PMC3814340; doi:10.1371/journal.pntd.0002480)
Supplement: Table S2 — Mortality of epilepsy from the included. (DOC) [file pntd.0002480.s005.doc]

**Table S2. Mortality of epilepsy from the included studies (N=5)**

| **Reference** | **Year** | **Country** | **PWE**  N age | | | **SMR** | **CI** |
| --- | --- | --- | --- | --- | --- | --- | --- |
| **Rural** | | | | | | | |
| Nicoletti 2009 | 1999-2009 | Bolivia | 103 | all | | 1.34 | 0.68-2.39 |
| **Urban** | | | | | | | |
| Devilat 2004 | 1996-2002 | Chile | 16 | children | | 3.21 | 1.48-4.95 |
| Carpio 2005 | 1997-2000 | Ecuador | 379 | all | | 6.3 | 2.0-10.0 |
| Kochen 2005 | 1990-1998 | Argentina | 96 | | all | 2.45 | / |
| Mota Gomez 2011 | 2004-2007 | Brasil | Population | | | 0.76 | / |

CI: confidence intervals; PWE: people with epilepsy; SMR: standardized mortality ratio.
