# Supplementary material for: Epilepsy and Neurocysticercosis in Latin America: A Systematic Review and Meta-analysis
Source: PLoS Negl Trop Dis. 2013 Oct 31;7(10):e2480. doi: 10.1371/journal.pntd.0002480 (PMC3814340; doi:10.1371/journal.pntd.0002480)
Supplement: Table S3 — Study Description of cysticerosis (CC) or neurocysticercosis (NCC) in Patients With Epilepsy (without control group). (DOC) [file pntd.0002480.s006.doc]

**Table S3. Study Description of Cysticerosis (CC) or Neurocysticercosis (NCC) in Patients With Epilepsy (without control group) (N=21)**

| **Reference** | **Year of Study** | **Country** | **Study**  **design** | **Participants**  **(Total)** | **Age** | **Age(years)**  **Range,mean,+SD** | **Source of patients With Epilepsy** | **Epilepsy Ascertainment** | **Epilepsy Definition/Classification** | **CC/NCC**  **Ascertainment** |
| --- | --- | --- | --- | --- | --- | --- | --- | --- | --- | --- |
| Arruda et al., 1991 | 1988-1990 | Brazil | Case-series | 210 | >10 years-old | (14-82)34.2+13.2 | Hospital based | CE+EEG | ILAE 1981 | CT  CSF Test and Surgery |
| Bucardo et al.,2005 | 1998 | Nicaragua | Case-series | 88 | All ages | (6-53)25.9+10.6 | Hospital based | CE | Not referred | Serum AbELISA  Serum EITB |
| Freitas et al., 2005 | 2001 | Brazil | Case-series | 110 | Adults | Not referred | Hospital based | CE | Not referred | Serum EITB  Serum AgELISA |
| Gaffo et al., 2004 | 1998-1999 | Peru | Prospective | 54 | Children | (2-14)9.3 | Hospital based | CE+EEG | ILAE 1981 | CT  Serum EITB (in CT+) |
| Garcia et al., 1999 | Not referred | Peru | Case-series | 16 | Adults | (17-56)29.5 | Hospital based | CE+EEG | Not referred | Serum EITB  CT (in EITB+) |
| Gomes et al., 2000 | 1998 | Brazil | Case-series | 200 | All ages | Not referred | General population | CE | WHO 1987 | Serum EITB  Serum AbELISA |
| Ishida et al., 2006 | Not referred | Brazil | Case-series | 88 | All ages | Not referred | Hospital based | CE | Not referred | Serum EITB  Serun AbELISA  CT |
| Medina et al., 2011 | 1997-2005 | Honduras | Prospective | 1121 | All ages | Not referred | General population | Q+CE+EEG | ILAE 1981,1989,1993 | CT |
| Medina et al., 2005 | 1997 | Honduras | Cross sectional | 6473 | All ages | Not referred | General population | Q+CE+EEG | ILAE 1981,1989 | Serum EITB  CT |
| Medina et al., 1990 | Not referred | Mexico | Case-series | 100 | Adults | (25-80)36.8 | Hospital based | CE+EEG | ILAE 1981 | CT |
| Montano et al., 2005 | 1999-2000 | Peru | Cross sectional | 913 | All ages | Not referred | General population | Q+CE | ILAE 1981,1989 | Serum EITB  CT |
| Nicoletti et al.,2005 | 1994 | Bolivia | Cross sectional | 9955 | All ages | Not referred | General population | Q+CE+EEG | ILAE 1981 | Serum EITB  CT |
| Palacio et al., 1998 | 1995 | Colombia | Prospective | 643 | Adults | 32.9+15.3 | Hospital based | CE | WHO 1993 | CT+EITB |
| Rigatti et al., 1999 | 1990-1998 | Brazil | Retrospective | 120 | Adults | Not referred | Hospital based | HR-CE | Not referred | CT |
| Rogel-Ortiz et al., 1999 | 1989-1997 | Mexico | Prospective | 130 | Adults | (21-87)45 | Hospital based | CE+EEG | ILAE 1981 | CT+MRI |
| Silva-Vergara et al., 1994 | 1992-1993 | Brazil | Cross sectional | 1080 | All ages | Not referred | General population | Q+CE | Not referred | CSF ELISA/RIFI  Serum ELISA/RIFI - CT |
| Suástegui et al., 2009 | 2000- | Mexico | Prospective | 455 | Adults | (20-89)43+16.9 | Hospital based | EEG | Not referred | CT |
| Trentin et al., 2002 | 1978-1990 | Brazil | Retrospective | 1000 | All ages | Not referred | Hospital based | HR | ILAE 1981 | CT |
| Trevisol et al., 1998 | 1995-1996 | Brazil | Retrospective | 58 | >10 years-old | Not referred | Hospital based | HR | Not referred | CT |
| Valença et al., 2000 | 1996-1999 | Brazil | Prospective | 249 | >12 years-old | Not referred | Hospital based | CE+EEG+CSF | Not referred | CTorMRI |
| Villaran et al., 2009 | 1999-2005 | Peru | Cohort | 817 | > 1 year-old | Not referred | General population | Q+CE | ILAE 1993 | CT and/or MRI  Serum EITB |

AbELISA: serum antibody enzyme-linked immunosorbent assay; AgELISA: serum antigen enzyme-linked immunosorbent assay; CC: cysticerosis; CSF: cerebrospinal fluid; CT: brain computed tomography; E: neurological evaluation; EITB: enzyme-linked immunoelectrotransfer blot assay; NCC: neurocysticercosis; MRI: magnetic resonance imaging; Q: questionnaire, RIA: immunofluorescence assay; T: tool (EEG: electroencephalography ; MR: medical records).
