# Supplementary material for: Epilepsy and Neurocysticercosis in Latin America: A Systematic Review and Meta-analysis
Source: PLoS Negl Trop Dis. 2013 Oct 31;7(10):e2480. doi: 10.1371/journal.pntd.0002480 (PMC3814340; doi:10.1371/journal.pntd.0002480)
Supplement: Table S4 — Studies on the association between cysticercosis/neurocysticerosis and epilepsy in Latin American Countries. (DOC) [file pntd.0002480.s007.doc]

**Table S4. Studies on the association between cysticercosis/neurocysticerosis and epilepsy in Latin American Countries (N=10)**

| ***Study*** | ***CC/NCC***  ***Ascertainment*** | ***PWE***  ***(n)*** | ***C/NCC in PWE*** | ***PWOE***  ***(n)*** | ***CC/NCC***  ***in PWOE*** | ***OR*** | ***95%CI*** | ***P*** |
| --- | --- | --- | --- | --- | --- | --- | --- | --- |
| **Bonametti 1992** | CSF AbELISA | 50 | 17 | 40 | 0 | Undefined |  | <0.001 |
|  | Serum AbELISA | 50 | 10 | 50 | 1 | 12.25 | 1.5-266.59 | <0.05 |
| **Correa 1999** | Serum AgELISA | 68 | 13 | 133 | 9 | 3.26 | 1.21-8.86 | <0.05 |
|  | Serum EITB | 68 | 15 | 133 | 17 | 1.61 | 0.7-3.7 | 0.21 |
| **Cruz 1999** | CT | 26 | 14 | 118 | 17 | 6.93 | 2.5-19.48 | <0.001 |
|  | Serum EITB | 28 | 6 | 96 | 10 | 2.35 | 0.67-8.07 | 0.12 |
| **Del Brutto 2005** | CT | 22 | 6 | 1664 | 139 | 4.11 | 1.41-11.41 | <0.05 |
|  | Serum EITB | 19 | 5 | 19 | 1 | 6.43 | 0.59-163-25 | 0.18 |
| **Garcia 1993** | Serum EITB | 189 | 22 | 306 | 8 | 4.91 | 2.04-12.41 | <0.001 |
| **Garcia 1997** | Serum EITB | 41 | 14 | 8 | 1 | 3.63 | 0.37-86.47 | 0.41 |
| **Garcia-Noval 1996** | CT | 76 | 36 | 51 | 12 | 2.92 | 1.25-6.96 | <0.05 |
|  | Serum EITB | 80 | 14 | 1462 | 165 | 1.67 | 0.87-3.13 | 0.13 |
| **Gracia 1990** | Serum EITB | 19 | 0 | 44 | 0 | Undefined |  |  |
| **Nicoletti 2002** | Serum EITB | 113 | 22 | 233 | 27 | 1.85 | 0.99-3.4 | 0.07 |
| **Schantz 1994** | CT | 10 | 7 | 109 | 15 | 14.62 | 2.93-81.87 | <0.001 |
|  | Serum AbELISA | 42 | 0 | 2482 | 52 | 0 | 0-5.57 | 0.4 |
|  | Serum EITB | 42 | 12 | 2482 | 202 | 4.51 | 2.15-9.34 | <0.001 |

AbELISA: serum antibody enzyme-linked immunosorbent assay; AgELISA: serum antigen enzyme-linked immunosorbent assay; CC: cysticerosis; CSF: cerebrospinal fluid; CT: brain computed tomography; EITB: enzyme-linked immunoelectrotransfer blot assay; NCC: neurocysticercosis; PWE: people with epilepsy; PWOE: people without epilepsy; PWE CC+: people with epilepsy affected by cysticercosis; PWOE CC+: people without epilepsy affected by cysticercosis.
