# Supplementary material for: Epilepsy and Neurocysticercosis in Latin America: A Systematic Review and Meta-analysis
Source: PLoS Negl Trop Dis. 2013 Oct 31;7(10):e2480. doi: 10.1371/journal.pntd.0002480 (PMC3814340; doi:10.1371/journal.pntd.0002480)
Supplement: Table S5 — Meta-regression of epilepsy incidence: univariate and multivariable analysis. (DOC) [file pntd.0002480.s008.doc]

**Table S5. Meta-regression of epilepsy incidence: univariate and multivariable analysis**

|  | **UNIVARIATE** | | | |  | **MULTIVARIABLE** | |
| --- | --- | --- | --- | --- | --- | --- | --- |
|  | **p- value** | **Odds ratio** | **Heterogeneity**  **(τ^2^)** | **Heterogeneity**  **(%)** | **p- value** | | **Odds**  **ratio** |
| **Study setting**  Urban  Rural | 0.5 | 1.0  0.6 | 0.04 | -18.3 |  | |  |
| **Age group**  All  Adults  Children | 0.6 | 1.0  1.2  0.5 | 0.03 | -22.2 |  | |  |
| **Epilepsy ascertainment** |  |  |  |  |  | |  |
| Q+E  Q+E+T | 0.6 | 1.0  0.5 | 0.04 | -22.9 |  | |  |
| **Study design**  Prospective  Retrospective | 0.2 | 1.0  1.4 | 0.02 | 33.4 | 0.6 | | 1.0  1.2 |
| **Study size**  >20,000  1,000-20,000  <1,000  **Definition of epilepsy**  ILAE 1993  Other definition  **Questionnaire**  WHO  Others  **Validated questionnaire**  Yes  No  **Administrators**  Health-care personnel  Others  **CC/NCC**  **≤**median prevalence*  >median prevalence* | 0.2  0.2  0.6  0.8  0.5  0.6 | 1.0  1.1  1.7  1.0  1.7  1.0  0.6  1.0  0.2  1.0  0.1  1.0  1.5 | 0.03  0.02  0.04  0.04  0.04  0.09 | 33.5  33.5  -18.3  -31.3  -22.9  -5.1 | 0.2  0.8 | | 1.0  1.2  1.7  1.0  1.5 |

CC: cysticercosis; E: neurological evaluation; NCC: neurocysticercosis Q: questionnaire; T: tool.

*median seroprevalence (EITB) estimates among people with epilepsy in Latin American countries: 11·6% for studies performed in urban areas, 25·0% for studies performed in rural areas.
