# Supplementary material for: Epilepsy and Neurocysticercosis in Latin America: A Systematic Review and Meta-analysis
Source: PLoS Negl Trop Dis. 2013 Oct 31;7(10):e2480. doi: 10.1371/journal.pntd.0002480 (PMC3814340; doi:10.1371/journal.pntd.0002480)
Supplement: Table S6 — Meta-regression of epilepsy treatment gap (TG): univariate and multivariable analysis. (DOC) [file pntd.0002480.s009.doc]

**Table S6. Meta-regression of epilepsy treatment gap (TG): univariate and multivariable analysis**

|  | **UNIVARIATE** | | | |  | **MULTIVARIABLE** | |
| --- | --- | --- | --- | --- | --- | --- | --- |
|  | **p- value** | **Odds ratio** | **Heterogeneity**  **(τ^2^)** | **Heterogeneity**  **(%)** | **p- value** | | **Odds**  **ratio** |
| **Study setting**  Urban  Rural | **0.01** | 1.0  **4.0** | 0.4 | 58.9 | **0.02** | | 1.0  **2.7** |
| **Age group**  All  Adults  Children | 0.2 | 1.0  0.9  1.1 | 0.8 | 2.8 | 0.6 | | 1.0  0.6  1.3 |
| **Study size**  >20,000  1,000-20,000  <1,000 | **0.03** | 1.0  **1.6**  **2.5** | 0.5 | 33.0 | 0.3 | | 1.0  1.7  2.8 |
